# Internalizing disorders rather than ADHD are risk factors for chronicity in pediatric migraine patients

**DOI:** 10.55730/1300-0144.5870

**Published:** 2024-07-12

**Authors:** Nevra ÖKSÜZ, Gülen Güler AKSU, Asena Ayça ÖZDEMİR, Aynur ÖZGE

**Affiliations:** 1 Department of Neurology, Faculty of Medicine, Mersin University, Mersin, Turkiye; 2 Department of Child and Adolescent Psychiatry, Faculty of Medicine, Mersin University, Mersin, Turkiye; 3 Department of Medical Education, Faculty of Medicine, Mersin University, Mersin, Turkiye

**Keywords:** Migraine, childhood, chronification, internalizing, externalizing, frontostriatal circuit

## Abstract

**Background/aim:**

Migraine is a prevalent neurological disorder that can lead to disability in children and adolescents. It is frequently accompanied by psychiatric comorbidities, both internalizing and externalizing disorders. While the relationship between migraine and internalizing disorders has been studied, there is limited research on the link between migraine and attention deficit hyperactivity disorder (ADHD).

**Materials and methods:**

A total of 280 pediatric headache patients, 107 with externalizing (ADHD) and 173 with internalizing disorders (depression and/or anxiety), were included. The dataset was composed using the Turkish headache database, Mersin Branch. Pain characteristics, associated symptoms, and accompanying comorbidities were evaluated retrospectively.

**Results:**

Two hundred four patients were followed up with episodic migraine (EM) and 76 patients with chronic migraine (CM). One hundred forty-six boys and 134 girls were evaluated, and internalizing disorders were more common in the girls (p < 0.001). It was a much more prominent accompaniment in chronic migraine internalizing disorders (p = 0.038). EM, on the other hand, was more frequent in ADHD. Pain intensity and frequency were greater in those with internalizing disorders (p = 0.007), while photophobia was more prevalent in those with ADHD (OR; 0.555, p = 0.044). Moreover, we observed that individuals with internalizing disorders were predominantly female (p = 0.003) and had a higher mean age (p < 0.001) than those with externalizing disorders.

**Conclusion:**

Internalizing disorders seem to be a risk factor for migraine chronification in pediatric migraine. ADHD is a prototypic externalizing disorder more associated with EM. This outcome provides an opportunity to follow our patients in terms of prognosis and offers us the chance for a better evaluation. Identifying factors that contribute to the chronicity of migraine may lead to better management and reduced disability for migraine sufferers.

## 1. Introduction

Primary headaches are the most common manifestation of pain in childhood and adolescents and have a significantly impact on children’s quality of life [1]. Migraine affects 9.1% of children and adolescents globally, making this condition a common concern for many patients and their doctors [2]. Migraine in children has a high risk of chronification and psychiatric comorbidities are an important risk factor for this process [3,4]. Psychiatric disorders can be classified as externalizing and internalizing disorders, although these terms are not used in the Diagnostic and Statistical Manual of Mental Disorders (DSM) [5,6]. Externalizing behaviors and disorders are mainly identified by actions in the outside world, such as hostility, aggression, acting out, and antisocial behavior. On the other hand, internalizing behaviors and disorders are mainly identified by processes that occur within oneself, such as depression, anxiety, and somatization. Externalizing disorders are characterized by observable behaviors, such as attention-deficit/hyperactivity disorder, oppositional defiant disorder, conduct disorder, antisocial personality disorder, and substance use disorders. Internalizing disorders, on the other hand, are characterized by inward and nonobservable processes, such as mood disorders (e.g., major depressive disorder and dysthymia) and anxiety disorders (e.g., generalized anxiety disorder, separation anxiety disorder, phobias, and obsessive–compulsive disorder). These terms are often used in research and clinical practice as a way to group similar disorders and understand the underlying factors. Although internalizing and externalizing psychopathology are separate based on their characteristic symptoms and behaviors, both of them can be seen at the same time in the same person [7].

Again, the early maladaptive schemas (EMSs) of these patients differ between internalizing and externalizing disorders. EMSs are deeply ingrained, self-defeating patterns of thoughts, feelings, and behaviors that develop during childhood as a result of negative experiences and innate temperament. These schemas are enduring and pervasive, influencing the individual’s perceptions of themselves, others, and the world around them. They often lead to negative emotions, maladaptive behaviors, and interpersonal problems, and can contribute to the development of various internalizing and externalizing disorders. For example, two EMSs, social isolation and vulnerability to harm/illness, explained 45% of the variation in internalizing problems. Furthermore, the EMSs of entitlement/grandiosity and dependence/incompetence accounted for 19% of the variation in externalizing problems, according to the same study [8]. EMSs clearly illustrate our thought patterns, such as coping strategies, and may be related to our temperament, characteristics of headaches, and our prognosis. Since different schemas are defined in internalizing and externalizing disorders and these schemas are considered to have an effect on migraine, internalizing and externalizing disorders should be investigated in detail in this respect [9]. Recent studies have also demonstrated that individuals with migraine and comorbid internalizing disorders exhibit frontostriatal circuit dysfunction, which plays a crucial role in the pathophysiology of these disorders [10,11]. Furthermore, it was reported that individuals with migraine had significantly reduced striatal dopamine transporter availability, indicating frontostriatal circuit dysfunction in migraine. It would be useful to look at patients from this perspective [12].

Internalizing and externalizing symptoms are important in chronicity associated with psychiatric comorbidities. A high level of internalizing and externalizing symptoms was found to be associated with a lower chance of remission in headache syndromes [13]. Internalizing disorder is focused on the own self, while externalizing disorder particularly occurs in interaction with the social environment. Internalizing symptoms primarily include anxiety and depressive symptoms, while externalizing symptoms include aggressive behavior, anger, and hyperactivity correlated with headache in children and adolescents [14]. We know that there is a bidirectional relationship between headaches and psychopathology in children and adolescents. Headache leading to psychiatric symptoms contributes to missed school days as well as poor cognitive functioning and family/friend relationships; conversely, the presence of psychopathology, especially internalizing disorders, leads to somatic complaints and headache [3]. Children with internalizing disorders are more stressed and introverted by nature. Therefore, it is thought that these children will have more somatic complaints and headaches, so its relationship with headaches has been studied more. Whereas previous research has reported associations between headaches and internalizing disorders, particularly depression and anxiety, recent research has shown that headaches are also common in externalizing disorders [15,16]. For example, migraine was found to be approximately 2.5 times more common in people with ADHD, which is an externalizing disorder [17]. The Epidemiology of Childhood Psychopathology in Türkiye (EPICPAC-T), the largest epidemiologic face-to-face survey of school-age children in Türkiye, found that ADHD is the most common disorder with (19.5%) and without impairment (16.7%) among 6- to 13-year-old school children. Again, oppositional deficient disorder (ODD) and conduct disorder (CD), types of disruptive behavior disorders, are frequently seen in children and adolescents at the rate of 3.44% and 0.36%, respectively [18]. Likewise, depression and anxiety are common in children, with a prevalence of 6.2% and 3.2%, respectively [19].

Due to the high incidence of these diseases and the high correlation between them and migraine, it becomes important to investigate the characteristics of migraine in these diseases. Since migraine has a high disease burden and causes severe disability, we thought that determining the characteristics of migraine would contribute to the management of headaches in these diseases and give an idea about its possible course. Based on this, we searched for possible clinical clues to migraine differences in internalizing and externalizing disorders. To add evidence to the preexisting research, here we aimed to describe the migraine characteristics in externalizing and internalizing disorders.

## 2. Materials and methods

### 2.1. Study design and data collection

This study was planned as a single-center, retrospective, observational, comparative case series. We included 173 patients with internalizing disorders and 107 patients with externalizing disorders among the headache patients, a total of 280 children and adolescents that we followed together between 2018 and 2022. The dataset was composed using the Turkish headache database, Mersin Branch. All patients’ information in this database was in detail.

In the present study, a child and adolescent psychiatrist conducted face-to-face psychiatric interviews with all patients and their parents. She asked for teacher observation forms to elaborate on psychiatric anamnesis if necessary. All psychiatric disorders were diagnosed according to DSM-IV diagnostic criteria by the same child and adolescent psychiatrist. According to the DSM-IV diagnostic criteria, patients with ADHD and/or oppositional and/or conduct disorder were classified as having externalizing disorders, while those with depression and/or anxiety disorders (generalized anxiety disorder, social phobia, panic disorder, and obsessive–compulsive disorder) were evaluated as having internalizing disorders. The study included 173 patients with internalizing disorders, including depression (n = 47), anxiety disorder (n = 68), obsessive–compulsive disorder (n = 32), and depression with anxiety (n = 26). The study also involved 107 patients with externalizing disorders, including ADHD (n = 77), ADHD with ODD (n = 23), and ADHD with CD (n = 7). We excluded from the study patients who had both internalizing and externalizing disorders. Patients with mental retardation, psychotic disorder, bipolar disorder, pervasive developmental disorder, alcohol and substance abuse, and tic disorder were also excluded.

The study participants were younger than 18 years of age and had migraine, including 46 cases of migraine with aura (MwA), 158 cases of migraine without aura (MwoA), and 76 cases of chronic migraine (CM). The classification of migraine was based on the International Classification of Headache Disorders, 3rd edition (Headache Classification Committee, 2018) [20]. Patients with a “headache-plus” diagnosis (e.g., migraine plus tension-type headache) were excluded from the study. All patients underwent a neurological examination following a complete psychiatric evaluation. During face-to-face interviews, we noted the presence of migrainous features (nausea, vomiting, photophobia, phonophobia, and aggravation by physical activity), osmophobia, headache frequency, presence of aura, localization, intensity of pain according to the visual analog scale (VAS), and attack duration. In addition to demographic data, we recorded the presence of psychiatric illness in their parents, the presence of stress, and the duration of education of the subjects.

### 2.2. Statistical analysis

For data entry and analysis, we used the program TIBCO Statistica version 13.5.0.17. The results are given as 95% confidence intervals and p < 0.05 was considered significant. We used the Shapiro–Wilk test to assess the normality of continuous variables. Nonparametric methods were used because continuous variables were not normally distributed. We used the Mann–Whitney U test for comparing two independent groups and the chi-squared test in the analysis of categorical data. Multiple logistic regression backward elimination was used for variables that may influence internalizing and externalizing disorders.

## 3. Results

Our study evaluated a total of 280 participants, including 146 boys (52.1%) and 134 girls (47.8%). There were differences between the patient groups regarding age and sex distribution for both boys and girls. Participants in the internalizing group were significantly older than those in the externalizing group (p < 0.001). Moreover, there was a higher proportion of girls in the internalizing group (p = 0.003). Additionally, participants in the externalizing group had slightly lower levels of education than those in the internalizing group (p = 0.015).

Regarding the presence of psychiatric disorders in parents, 164 patients had data on this matter. The mother and/or father of 53 patients had a history of psychiatric disorder and was significantly higher in externalizing disorders (p = 0.041). Further details on the demographic and headache characteristics of the patients are shown below (Table 1).

When examining the characteristics of headaches, we found that participants with internalizing disorders had significantly higher levels of pain severity and headache frequency (p = 0.001, p = 0.007) in comparison to those with externalizing disorders as shown in Table 2. However, there was no significant difference between the two groups in terms of pain duration (p = 0.097) or aura. The prevalence of MwoA was significantly higher in the externalizing group, while CM was significantly higher in the internalizing group (p = 0.038). The migraine characteristics and related factors are summarized below (Table 2).

Our results indicated that there was no significant difference in the distribution of most symptoms between the two groups, including nausea; vomiting; and light, sound, and smell sensitivity, as well as physical activity. Similarly, there was no significant difference in pain quality (p = 0.169) or in the distribution of pain (unilateral or bilateral headache, p = 0.602) between the two groups. Stress and physical activity, which are among the most common triggers, did not differ significantly between the groups (p = 0.074 and p = 0.492, respectively).

Age, sex, nausea, vomiting, photophobia, phonophobia, osmophobia, stress, physical activity, pain frequency, pain duration, pain intensity, localization, and throbbing pattern were included in the multiple regression model to investigate the relationship between internalizing and externalizing disorders. By removing the nonsignificant variables according to the backward elimination, a meaningful model was formed with the remaining variables in Table 3.

The results revealed that photophobia had an odds ratio (OR) of 0.555 (95% CI: 0313–0.984) and a p-value of 0.044 for having internalizing disorders. This suggests that individuals with externalizing disorders were more likely to experience photophobia than those with internalizing disorders. Additionally, the severity of pain and headache frequency had positive associations with internalizing disorders, with ORs of 1.370 (95% CI: 1.124–1.670) and 1.036 (95% CI: 1.003–1.071), respectively (p = 0.002; p = 0.033). The results also indicated that being female and increasing age were associated with internalizing disorders (p = 0.008; p = 0.009). These variables, which differed significantly as a result of our study, are summarized in Figure, which displays clinical clues regarding the course of migraine in internalizing or externalizing disorders.

Our study findings suggest that internalizing disorders may be a risk factor for chronification in pediatric migraine. Being female and increasing age also appear to be risk factors for this chronification.

## 4. Discussion

Migraine is a common neurological disorder that affects children and adolescents, often causing significant disability. Individuals with migraine may also experience comorbid psychiatric conditions, such as internalizing and externalizing disorders. While research has explored the association between migraine and internalizing disorders, there is limited understanding of the relationship between migraine and externalizing disorders, including ADHD. To address this gap, we conducted a study aiming to better understand the characteristics of migraine in these conditions and identify factors contributing to migraine chronification. Our study findings shed light on the associations between migraine and internalizing and externalizing disorders, providing important insights for the coping with and treatment of migraine in these patients.

Most of the cross-sectional studies conducted to date have revealed an association between migraine and many diseases, such as anxiety disorders and depression [21]. However, the underlying mechanisms of the relationship between migraine and psychiatric disorders are still unclear. In recent years, the relationship between migraine and externalizing disorders has started to attract attention [16,17].

In our study, we investigated whether there was a difference between internalizing and externalizing disorders in terms of migraine characteristics. We found that CM was more common in individuals with internalizing disorders, while EM, especially MwoA, was more common in those with externalizing disorders (p = 0.038). From the literature, it is apparent that psychiatric disorders are more common in patients with CM than in those with EM, and they increase the risk of chronic headaches and transformed migraine [21]. Similarly, CM was common in our study group (27.1%). The estimated prevalence of chronic daily headaches in children and adolescents ranges from 0.9% to 7.8% in studies conducted in several countries [22]. The high prevalence of CM in our study may have been due to comorbid psychiatric disorders or the fact that we are a specialized headache clinic.

Previous studies have shown that internalizing disorders, in particular, lead to somatic complaints and headaches [3]. In our study, pain intensity and frequency were found to be riskier in individuals with internalizing disorders than in those with externalizing disorders, as per multiple regression analysis. This suggests that the course of pain is more severe in internalizing disorders and that these disorders contribute to chronicity. Additionally, our study found that the prevalence of female sex was higher in individuals with internalizing disorders, which is consistent with the literature [6]. Although there was no difference in terms of migraine characteristics when they were compared individually, photophobia was higher in individuals with externalizing disorders when multiple regression analysis was performed.

When the genetic load is considered in terms of biological origin, externalizing disorders are more hereditary, while the genetic load of depression and anxiety is relatively less. Externalizing disorders ADHD, ODD, and CD are more long-term disorders with congenital features, while the behavioral aspect predominates, whereas internalizing disorders are disorders with a predominant cognitive aspect that develop later due to cognitive distortions [23]. The fact that the history of psychiatric disorder in the parents was significantly higher in the externalizing group in our study also supports these data (p = 0.041).

Externalizing disorders are chronic problems that negatively affect school life, home life, and peer relations for the child and family and are unnoticed but create great stress. It is known that, as a result of this chronic stress, internalizing disorders such as depression and anxiety disorders are added to the process in the long term, and the frequency of comorbidity increases, especially in adolescence [24]. In the present study, it was noteworthy that age and frequency of CM were higher in the internalizing disorder group (p = 0.009 and p = 0.038, respectively). Although we excluded the diagnosis of externalizing disorder in the group with internalizing disorder in the study, longitudinal follow-up studies of individuals with migraine and externalizing disorder at a young age may be informative in terms of defining the determinants of CM.

There is limited research exploring the relationship between migraine and externalizing disorders such as ADHD, although promising developments have been identified with regards to their association with frontostriatal circuit dysfunction [25]. Identifying the neurobiological mechanisms underlying these comorbidities and understanding the association between frontostriatal circuit dysfunction and migraine comorbidities are essential for optimal management and treatment of these conditions [10]. In this context, evaluating migraine characteristics in externalizing and internalizing disorders and identifying the factors that contribute to the chronicity of migraine disorders is a crucial step towards better understanding the neurobiological mechanisms of these conditions [26].

Although our study shed light on the differences in migraine characteristics between individuals with internalizing and externalizing disorders, there are some limitations that need to be considered. The study only included a relatively small sample size of 280 participants, which may not be representative of the entire population of children and adolescents with migraine and comorbid psychiatric disorders. Moreover, the study was conducted at a specialized headache clinic, which may have resulted in a selection bias towards more severe or chronic cases of migraine. We also did not include a control group of children and adolescents without migraine or psychiatric disorders, which would have enabled a better comparison of migraine characteristics between the different groups. Finally, the study did not investigate the potential impact of medication use on migraine characteristics in the different groups, which may have influenced the results.

The implications of our study highlight the importance of understanding the characteristics of migraine in both internalizing and externalizing disorders, which can aid in the management and treatment of these comorbidities. It is worth noting that the multidisciplinary approach used in the study, which involved both neurology and child/adolescent psychiatry departments, enabled a more comprehensive evaluation of migraine patients with comorbid psychiatric disorders. Furthermore, the comprehensive dataset used, which included detailed medical histories, physical examinations, and neuroimaging studies, provided a more thorough understanding of the characteristics of migraine in individuals with both internalizing and externalizing disorders. This highlights the importance of utilizing a multidisciplinary and comprehensive approach in future studies on migraine and psychiatric comorbidities. Additionally, the design of the study focused on the frontostriatal circuit, a neural network that is known to be involved in a wide range of cognitive and emotional functions, including reward processing, decision-making, and impulse control. By considering the frontostriatal circuit in the context of migraine comorbidities, our study provides a more comprehensive understanding of the neurobiological mechanisms underlying these conditions.

In conclusion, the high prevalence of psychiatric disorders in children and adolescents with migraine highlights the need for understanding the headache characteristics and assessing the risk of chronicity in terms of treatment and prognosis, particularly for children and adolescents. The present study provides insights into the differences in migraine characteristics between internalizing and externalizing disorders and emphasizes the necessity for further research in this field. Furthermore, the association between frontostriatal circuit dysfunction and migraine comorbidities underscores the importance of investigating the underlying neurobiological mechanisms to enhance the management and outcomes of patients with these conditions. Our study is important in that it is the first to address the characteristics of migraine in internalizing and externalizing disorders. These findings may lead to a reduction in disability and an improvement in the quality of life of affected individuals.

## Figures and Tables

**Figure:**
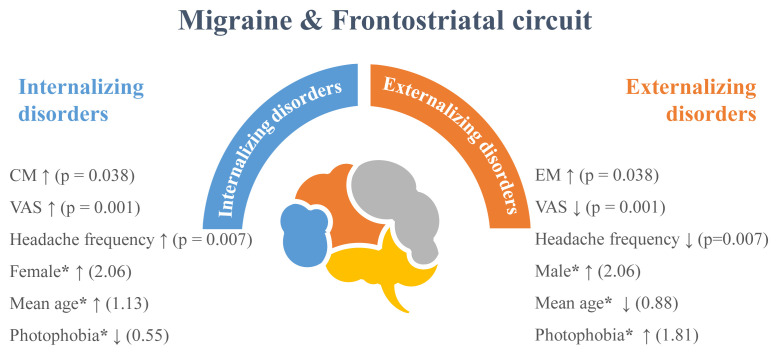
Clinical clues of migraine in internalizing or externalizing disorders. CM: chronic migraine, EM: episodic migraine, VAS: visual analog scale. *Risk coefficients were determined by multiple regression analysis.

**Table 1:** Clinical and demographic characteristics of the study patients.

		Internalizing disorders (n: 173)		Externalizing disorders (n: 107)		
		Mean ± SD (Min–Max)	Median [IQR]	Mean ± SD (Min–Max)	Median [IQR]	p
**Age**		14.52 ± 2.82 (4–18)	15 [13–17]	13.2 ± 2.79 (6–18)	13 [12–15]	**<0.001** a
**Education period (years)**		6.68 ± 3.3 (0–12)	7 [4–9]	5.81 ± 2.76 (0–11)	6 [4–7]	**0.015** a
**Sex**	**Male**	78	45.1	68	63.6	**0.003** b
	**Female**	95	54.9	39	36.4	
**Psychiatric disorder in parents**		12	21.8	41	37.6	**0.041** b

a Mann–Whitney U test,

b Chi-squared test.

**Table 2:** Migraine phenotypic features of the study patients.

		Internalizing disorders (n: 173)		Externalizing disorders (n: 107		
		Mean ± SD (Min–Max)	Median [IQR]	Mean ± SD (Min–Max)	Median [IQR]	p
**Headache frequency (days/month)**		12.76 ± 8.64 (1–30)	10 [6–16.5]	10.11 ± 7.75 (1–30)	8 [4–15]	**0.007** a
**Attack duration (minutes)**		296.07 ± 351.91 (30–1800)	150 [60–360]	222.24 ± 264.83 (30–1440)	120 [60–240]	0.097^a^
**VAS**		7.51 ± 1.41 (4–10)	7 [7–9]	6.96 ± 1.27 (3–10)	7 [6–8]	**0.001** a
		n	%	n	%	p
**Subtypes**	**MwA**	28	16.2	18	16.8	**0.038** b
	**MwoA**	89	51.4	69	64.5*	
	**CM**	56	32.4*	20	18.7	
**Headache quality**	**Pressing**	39	22.5	32	29.9	0.169^b^
	**Throbbing**	134	77.5	75	70.1	
**Localization**	**Unilateral**	98	56.6	64	59.8	0.602^b^
	**Bilateral**	75	43.4	43	40.2	
**Aura**		47	27.2	19	17.8	0.071^b^
**Nausea**		114	65.9	71	66.4	0.937^b^
**Vomiting**		49	28.3	21	19.6	0.102^b^
**Photophobia**		109	63.0	71	66.4	0.570^b^
**Phonophobia**		91	52.6	58	54.2	0.794^b^
**Osmophobia**		63	36.4	30	28.0	0.148^b^

* represents a significantly higher rate,

a Mann–Whitney U test,

b Chi-squared test,

MwA: migraine with aura, MwoA: migraine without aura, CM: chronic migraine, VAS: visual analog scale.

**Table 3:** Predictor variables and their association with internalizing disorders.

	Odds ratio	95% C.I.		p
		Lower	Upper	
Sex	2.058	1.210	3.499	**0.008**
Age	1.131	1.032	1.240	**0.009**
Photophobia	0.555	0.313	0.984	**0.044**
Headache frequency (days/month)	1.036	1.003	1.071	**0.033**
VAS	1.370	1.124	1.670	**0.002**
Constant	0.022			0.000

p: multiple logistic regression.
